# Physical mechanism and modeling of heat generation and transfer in magnetic fluid hyperthermia through Néelian and Brownian relaxation: a review

**DOI:** 10.1186/s12938-017-0327-x

**Published:** 2017-03-23

**Authors:** Suriyanto, E. Y. K. Ng, S. D. Kumar

**Affiliations:** 1 Nanyang Institute of Technology in Health and Medicine, Interdisciplinary Graduate School, Nanyang Technological University, Research Techno Plaza, #02-07, 50 Nanyang Drive, Singapore, 637553 Singapore; 2 Lee Kong Chian School of Medicine, Nanyang Technological University, Experimental Medicine Building, Level 3, Yunnan Garden Campus, 59 Nanyang Drive, Singapore, 636921 Singapore; 3 School of Mechanical and Aerospace Engineering, College of Engineering, Nanyang Technological University, 50 Nanyang Avenue, Singapore, 639798 Singapore

**Keywords:** Magnetic fluid hyperthermia, Nanotechnology, Computational modeling, Numerical methods, Bioheat transfer, Optimization

## Abstract

Current clinically accepted technologies for cancer treatment still have limitations which lead to the exploration of new therapeutic methods. Since the past few decades, the hyperthermia treatment has attracted the attention of investigators owing to its strong biological rationales in applying hyperthermia as a cancer treatment modality. Advancement of nanotechnology offers a potential new heating method for hyperthermia by using nanoparticles which is termed as magnetic fluid hyperthermia (MFH). In MFH, superparamagnetic nanoparticles dissipate heat through Néelian and Brownian relaxation in the presence of an alternating magnetic field. The heating power of these particles is dependent on particle properties and treatment settings. A number of pre-clinical and clinical trials were performed to test the feasibility of this novel treatment modality. There are still issues yet to be solved for the successful transition of this technology from bench to bedside. These issues include the planning, execution, monitoring and optimization of treatment. The modeling and simulation play crucial roles in solving some of these issues. Thus, this review paper provides a basic understanding of the fundamental and rationales of hyperthermia and recent development in the modeling and simulation applied to depict the heat generation and transfer phenomena in the MFH.

## Background

According to World Health Organization (WHO), there were 14 million incidences of cancer and 8.2 million of cancer associated deaths in 2012. In addition, the new cases of cancers would increase by 70% over the next 2 decades [1]. Currently, the clinically available cancer treatments are chemotherapy, radiotherapy, and surgical excision. However, there are several drawbacks in using these treatment modalities. In chemotherapy, chemotherapeutic drugs are injected into the body for the elimination of cancerous cells. Chemotherapy not only kills cancerous cells but it also has side effects such as lowering the immunity and increasing the risk of infection, inducing problems in the digestive system and also damaging the nervous system. Another treatment method, radiotherapy, uses radiation energy to destroy the tumor. Because it is hard to target the whole tumor, the radiation cannot eradicate the entire tumor effectively and the organs located around the targeted radiation region may lose their functionality. Surgery can be successful only if the exact cancer location can be approached. In the other words, the surgical option is not available for deep-seated tumors. Furthermore, surgery may induce the risk of infection and also cause complications on surrounding organs.

Due to the drawback of currently accepted cancer treatment modalities, alternative or adjunctive therapies have been sought after by clinicians. One of the promising treatment modalities is thermotherapy where heat is used to kill the cancerous cells. There are 2 major types of heating used in thermotherapy, namely hyperthermia and thermal ablation [2]. Hyperthermia is a thermotherapy where heat is used to increase the whole body or part of the body temperature from a normal body temperature of 37 °C to the temperature range of 41–45 °C. The heating of tumor to this temperature is used to enhance the therapeutic effect of radiotherapy and chemotherapy. This heating has the potential to selectively kill cancerous cells without harming surround healthy tissue [3]. Thermal ablation, as suggested by the term ‘ablation’, refers to the application of a high temperature, above 45 °C, with the aim to produce significant tissue destruction. This heating will cause cell death of both tumor and surrounding tissues, hence it has to be applied with caution [4].

During hyperthermia, the temperature of the tumor is increased to around 42 °C for a duration of an hour or longer [5, 6]. This applied heat changes the biomolecular systems of cells [7–10] and leads to cell death due to either apoptosis or necrosis [11–13]. There are strong biological rationales in accepting hyperthermia as a cancer therapy method [14, 15]. One of the fundamental reasons for using hyperthermia as a cancer therapy method is the discovery that the tumor is more sensitive to heating than healthy tissues. It implies that if the tumor is heated to 41–45 °C, the tumor will be destroyed without harming surrounding healthy tissues [16–18]. The tumor sensitivity to heat is due to the high acidity of the cancer cells as a consequence of the high glycolytic activities inside cancer cells [19]. Furthermore, the heat dissipated by blood flow protects the healthy tissue from overheating. As the temperature increases, the thermoregulation system of the body will remove the excess heat by increasing the blood flow; hence the blood volumetric flow rate is a function of temperature. In response to temperature rise, the blood flow rate in healthy tissues can increase up to 20 times higher than its preheating rate, while the heating would only cause an increase in the tumor blood flow rate up to double its flow rate [20, 21].

**Fig. 1:**
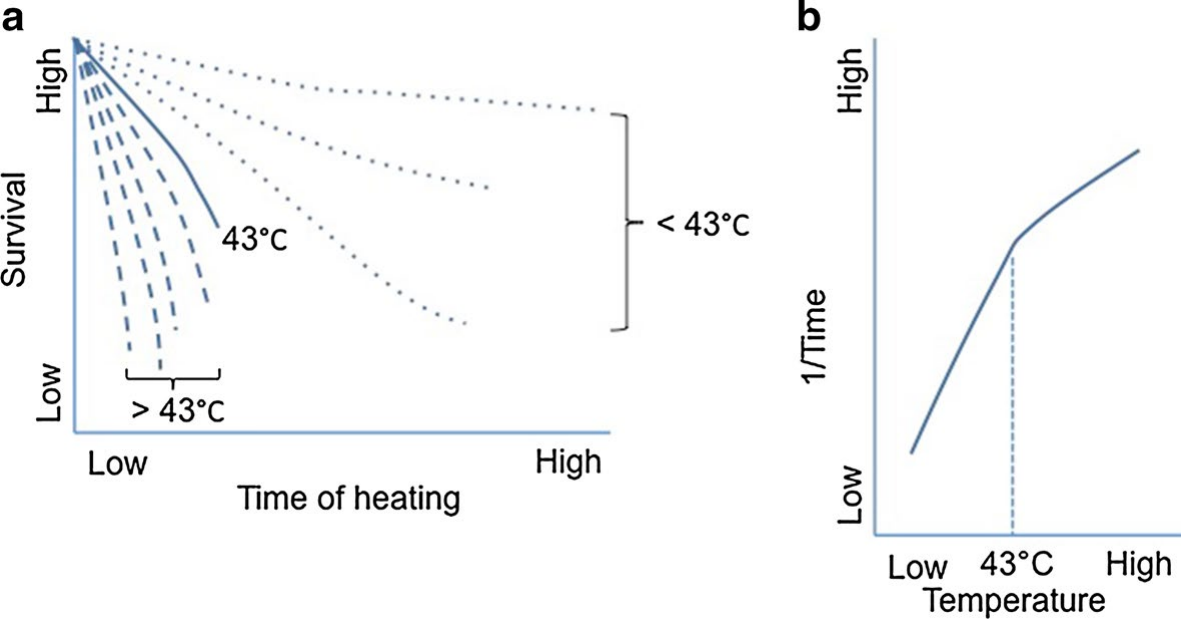
**a** Typical graph of survival curve of a cell line which is heated at different temperatures for various durations, **b** typical graph of Arrhenius plot with a break point at around 43 °C

The rate of cell death induced by heating in hyperthermia is dependent on the temperature and duration of treatment [22, 23]. Overgaard and Overgaard [24] carried out a study on the application of heat treatment on mouse mammary carcinoma to find out the relation between the temperature and time and suggested that longer heat exposure was required where a lower temperature of treatment was used, and vice versa [24]. The combination of these two parameters can be used to define the minimum heating time required to achieve irreversible damage on a tumor at a specific temperature setting [25]. This combination is expressed in the form of the thermal isoeffect dose which is the equivalent time–temperature heating setting that can generate the same heating outcome [26]. The isoeffect dose can be estimated using an Arrhenius plot. The Arrhenius plot depicts the time–temperature relationship of cells survival phenomenon due to applied heat is shown in Fig. 1. Factors affecting the Arrhenius plot include the thermotolerance, pH, cell phase in a cell cycle, and cell lines [27]. Generally, there is a breakpoint in the Arrhenius plot at around 43 °C which could be due to the thermotolerance of a patient in a prolonged heating of mild hyperthermia. The rate of cell death is double for every degree in temperature rise beyond the breakpoint of the Arrhenius plot. This killing rate decreases four to six times for every drop in temperature below the breakpoint [28, 29].

Commonly used methods to determine the thermal isoeffect dose is the cumulative equivalent minutes at 43 °C (CEM43 °C) which is a method for the conversion of heating duration at a specific temperature to an equivalent minute required for heating at 43 °C that generates the same heating outcome [30]. CEM43 °C can be used for comparing various hyperthermia treatment outcomes in different studies. There are variations of CEM43 °C such as CEM43 °C *T*_90_, and CEM43 °C *T*_50_. *T*_90_ refers to the magnitude of temperature reached and exceeded by 90% of the measuring point. Similarly, *T*_50_ refers to the magnitude of temperature reached and exceeded by 50% of the measuring point. Dewey [27] suggested that CEM43 °C *T*_90_ of around 25 and CEM43 °C *T*_50_ of around 400 were suitable for the hyperthermia treatment.

Other than using hyperthermia as a single cancer treatment modality, it can also be combined with other therapies to form multimodality treatment [31]. The combinations of hyperthermia and other cancer treatments modalities are able to provide a superior treatment outcome. One of the combinations is the use of hyperthermia and chemotherapy where heat can enhance the cytotoxicity of chemotherapeutic drugs [3, 32–36]. Through laboratory and clinical studies, Woodhall et al. found that better regression of tumors occurred in the treatment using this combination [37]. Similarly, in a number of studies, a synergetic effect of the combined treatment was observed and caused significant deaths of cancerous cells [38, 39]. In addition, Shingleton proposed that heating might increase the effectiveness of chemotherapy by increasing the local blood supply and oxygenation of tissues in response to body thermoregulation in removing excessive heat which enhanced the uptake of chemotherapeutic drugs in the tumor [40]. An in vivo animal study using this combination of treatment was conducted by Kossatz et al. [41] on breast tumor bearing nude mice. The result showed a significant reduction in tumor growth and complete tumor regression was observed in a number of cases.

Beside the multimodality of hyperthermia together with the chemotherapy, the tumor can also be treated using the synergetic effect of hyperthermia and radiotherapy [42]. Heating increases the cells sensitivity to radiation [43, 44]. This effect occurs because of the increase of blood flow that drives the tissue oxygenation enrichment and the momentary enhancement of radio sensitivity by facilitating formations of oxygen radical [3, 45, 46]. The enhancement of oxygenation in a tumor has been proved in the clinical trial done by Brizel et al. [47]. Furthermore, in this type of multimodality, the hyperthermia can suppress the healing mechanism that repairs damaged cells in the tumor [48, 49]. This damage on the repair system was further proved in the experiment done by Xu et al. [50] which suggested that the hyperthermia delocalized the DNA repair protein which led to an increase in the efficiency of radiotherapy. A number of preclinical and clinical trials on this multimodality model have been carried out [51–54]. An animal model test to examine the efficiency of hyperthermia in treating prostate cancer using 96 Copenhagen rats was done by Johannsen et al. The multimodality using hyperthermia and radiotherapy at a low radiation dose of 20 Gy was found to be able to produce the same therapeutic effect as using merely a radiation dose of 60 Gy [51]. Another animal model was done to prove the efficiency of applying hyperthermia for treatment of malignant glioma. The results showed a positive antitumor effect of hyperthermia on malignant brain tumors. Subsequently, clinical trials were performed to combine hyperthermia and radiotherapy for treating patients with glioblastoma multiforme [52, 53]. These trials showed that the target hyperthermia temperature could be achieved and the thermotherapy was a safe treatment modality since the therapeutic effect of hyperthermia had increased the overall survival rate of patients.

Advancement of thermotherapy required the involvement of experts from various fields including physicians, biologists, chemists, physicists, engineers, etc. This is due to the multidisciplinary scope in the development of thermotherapy and the transition process from bench to bedside. Some major technical concerns in hyperthermia have to be solved, including the planning, execution, monitoring and optimization of the treatment. This paper focuses on the application of magnetic nanoparticle heating for hyperthermia, specifically on the heat generation process through Néelian and Brownian relaxation and the recent advancement in the modeling and simulation of hyperthermia through these relaxation processes.

## Magnetic fluid hyperthermia

In hyperthermia, heat can be generated through different methods, such as the application of ultrasound [55, 56], microwave radiation [57], radiofrequency heating [58, 59], and laser photocoagulation [60]. These heating techniques have several common drawbacks that hinder their clinical acceptance. The waves generated by those methods cause unintended hotspots because there is a difficulty for the waves to focus on targeted areas. In addition, the waves have a low penetration ability [61, 62]. It is also hard to accurately raise and control the temperature of small tumors or deep-seated tumors using these methods [63]. Magnetic fluid hyperthermia (MFH) offers a solution to tackle the issues.

The magnetic nanoparticles have a wide range of applications in the biomedical sector [5, 64–67]. The use of MFH as a cancer therapy can be traced back to 1957 in a study carried out by Gilchrist et al. [68]. In their work on an animal model, MFH was concluded as a compelling method for the treatment of lymph nodes. At present, a number of studies have shown that the MFH alone could generate enough power to cause a complete regression of a tumor [69–71]. The use of MFH offers the solution to solve the hotspots and targeting issues that occur in other hyperthermia heating methods. The unwanted hot spots formation in the non-targeted area can be avoided in MFH because the use of nanoparticles offers a better control of energy deposition in the tumors by heating only the vicinity of particles [72]. For targeting issues, the high surface to volume ratio of magnetic nanoparticles enables the functionalization of particle surface with cancer-targeting molecules where a lot of researches are still ongoing to identify the suitable coating materials [73]. In addition, the targeting process can also be guided by the magnetic particle imaging [74, 75]. Besides, MFH offers other benefits, as hyperthermia is a minimally invasive treatment and the applied heat can be controlled therefore it minimizes the side effects. Besides, it can be used to treat complex geometries tumors. It can be used for treating the tumors hidden in vital organs where a surgical excision is not feasible [61]. The MFH has been assessed in a number of pre-clinical and clinical trials. An animal model was designed by Johannsen et al. to test the prospect of MFH in treating prostate cancer [76]. In their study, 48 male Copenhagen rats were treated with hyperthermia with a magnetic field frequency of 100 kHz and a field strength of 0–18 kA m^−1^. The results showed a significant difference in the tumor weight between the treated and control group. A follow-up prospective clinical trial on humans was conducted where 10 patients with locally recurrent prostate cancers underwent 6 thermal therapies [77]. In this study, the applied heat was found to be tolerable by the patients and the heating method using nanoparticles was successful. Besides, for the treatment prostate cancer, the use of MFH has been tested in for the treatment of different types of cancer on animal models. In a study on treating breast and pancreatic cancer using MFH, positive treatment outcome was obtained where a decline in the cancer cell proliferation and an increase in the apoptosis and necrosis were observed [78]. Another study on the rat breast carcinoma model showed that hyperthermia could inhibit tumor growth by downregulating the vascular endothelial growth factor and inhibiting the angiogenesis process in the tumor [79].

Magnetic nanoparticles can be delivered to the tumor through four different approaches, i.e. the arterial injection, the direct injection into the tumor region, the in situ implant formation using entrapped nanoparticle gel, and the active targeting by coating with the targeting ligands [80]. After the delivery of magnetic nanoparticles, an alternating magnetic field is activated to generate heat in the nanoparticles through an energy conversion based on the principle of the hysteresis loss or the Néelian and Brownian relaxation. Subsequently, heat dissipates to the surrounding tissues and causes the death of cancerous cells.

## Heat generation model based on Néelian and Brownian relaxation

**Fig. 2:**
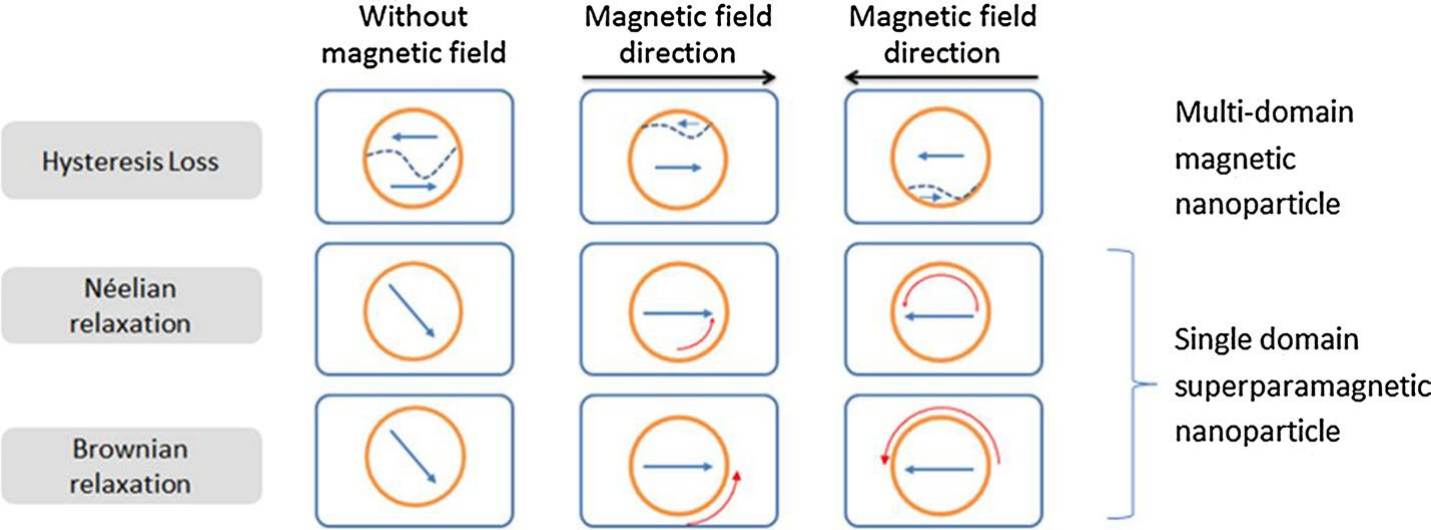
Different heat generation models in a magnetic nanoparticle in response to the alternative magnetic field. The *short straight arrows* represent the magnetic moment direction, the *curved arrows* represent the movement or change in direction, and the *dash lines* represent the domain boundaries in multi-domain particles

The heating in magnetic particles caused by changes of the magnetic field can occur via several mechanisms. The most well-known heating mechanism is by generating heat loss due to induced eddy currents which occur in bulk materials. Eddy currents heating occur due to the induced currents which are the flows of electrons against the electrical resistance of the material. For nano-sized materials, the eddy current heating is extremely weak since magnetic nanoparticles have a rather poor electrical conductivity to produce a noticeable induction heating and the nano-sized dimension of particles cannot generate a substantial electrical voltage [81]. For particles having a size of micro or nano-scale, the heating due to the hysteresis loss is dominant. Heat is generated by thermal energy dissipated through the magnetic domain reversal process that occurs in the ferromagnetic material. Especially for nanoparticles with a particle size smaller than its superparamagnetic critical diameter, the hysteresis loss is presented in terms of the Néelian and Brownian relaxation. The Néelian relaxation refers to the heating due to the energy loss produced by the rotation of individual magnetic moments within the particles. On the other hand, the Brownian relaxation refers to the heat generated by the physical rotation of particles due to the alignment process of magnetic moments with the external applied magnetic field. In MFH, the heat dissipated by nanoparticles is caused by the hysteresis loss as depicts in Fig. 2. This paper focuses on the heating process in superparamagnetic nanoparticles and hence, the discussion is limited to the heat generation due to the Néelian and Brownian relaxation which is the dominated heat generation process in nano-sized particles. More detailed information about these different types of heating mechanisms can be found in refs [82–86].

The modeling of heat generation and transfer process in MFH will increase the understanding of MFH phenomena and allow a successful transition of this technology from bench to bedside. In addition, the modeling through simulation can be used in the planning of the treatment and it also serves as a new alternative method for temperature mapping due to the difficulty in the real time temperature measurement during the treatment [87, 88].

The superparamagnetic nanoparticles are nano-sized particles with single-domain configuration due to the energetically instability of domain wall [89]. The use of these particles for hyperthermia offers benefits of allowing better dispersion of particles and avoiding the formation of particle aggregations. In order to qualify as a superparamagnetic nanoparticle, the particle’ diameter has to be smaller than its superparamagnetic critical diameter. These nano-sized particles exhibit a narrow hysteresis and the contributor to heat rise is the heating via Néelian and Brownian relaxation [90]. Rosensweig developed the formulation for the heat generation by superparamagnetic nanoparticles [91]. The equations for these two relaxation processes are given in Table 1 Eqs. 1, 2 and Table 1 Eq. 3 respectively. When Néelian and Brownian relaxations occur together, by using Eq. 4 Table 1 the effect of the combinatory process can be found. The volumetric power dissipation by monodispersed nanoparticles due to the applied magnetic field can be expressed as Table 1 Eqs. 5–8. These formulas are applicable when the susceptibility is assumed to be independent of the applied magnetic field. The power dissipated is often formulated in term of the specific loss power (SLP) or specific absorption rate (SAR), which is presented in Table 1 Eq. 9. When polydispersed particles are used, the heating power can be obtained by summarizing the heat generated from all particles as shown Table 1 Eqs. 10 and 11. Physical properties of potential materials used in MFH are provided in Table 2.

**Table 1:** Equations used in determining the heating power

Eq. no.	Parameter	Equation expression	Nomenclature
1	Néelian relaxation time, $\tau_{N}$ (s)	$\tau_{N} = \frac{\sqrt \pi }{2}\tau_{0} \frac{\exp \varGamma }{{\varGamma^{1/2} }}$	$\tau_{0}$ time constant (s) $K$ anisotropy constant (J m^−3^) $V_{M}$ magnetic volume (m^3^) $k_{B}$ Boltzmann constant $T$ absolute temperature (K)
2	Gamma, $\varGamma$	$\varGamma = \frac{{KV_{M} }}{{k_{B} T}}$	
3	Brownian relaxation time, $\tau_{B}$ (s)	$\tau_{B} = \frac{{3\eta V_{H} }}{{k_{B} T}}$	$\eta$ dynamic viscosity of the fluid (Pa s) $V_{H}$ hydrodynamic volume (m^3^)
4	Effective relaxation time, $\tau$ (s)	$\frac{1}{\tau } = \frac{1}{{\tau_{N} }} + \frac{1}{{\tau_{B} }}$	
5	Volumetric power dissipation, $P$ (W m^−3^)	$P = \pi \mu_{0} \chi_{0} H_{0}^{2} f\frac{2\pi f\tau }{{1 + (2\pi f\tau )^{2} }}$	$\mu_{0}$ free space permeability $H_{0}$ magnetic field strength (A m^−1^) $f$ magnetic field frequency (s^−1^) $\phi$ nanoparticles volume fraction $M_{d}$ domain magnetization (A m^−1^)
6	Equilibrium susceptibility, $\chi_{0}$	$\chi_{0} = \chi_{i} \frac{3}{\xi }(\coth \xi - \frac{1}{\xi })$	
7	Initial susceptibility, $\chi_{i}$	$\chi_{i} = \frac{{\mu_{0} \phi M_{d}^{2} V_{M} }}{{3k_{B} T}}$	
8	Langevin parameter, $\xi$	$\xi = \frac{{\mu_{0} M_{d} H_{0} V_{M} }}{{k_{B} T}}$	
9	Specific loss power (W kg^−1^)	$SLP = \frac{P}{\rho \phi }$	$\rho$ nanoparticle density (kg m^−3^)
10	Volumetric power dissipation of a polydispersion, $\bar{P}$ (W m^−3^)	$\bar{P} = \mathop \smallint \limits_{0}^{\infty } Pg\left( D \right)dD$	$D$ particle diameter (m) $\ln D_{0}$ median of $\ln D$ $\sigma$ standard deviation of $\ln D$
11	Particle size distribution function $g\left( D \right)$	$g\left( D \right) = \frac{1}{{\sqrt {2\pi } \sigma D}}\exp \left[ {\frac{{ - \left( {\ln \left( {D/D_{0} } \right)} \right)^{2} }}{{2\sigma^{2} }}} \right]$

**Table 2:** Physical properties of potential magnetic particles used in hyperthermia

Material	Superparamagnetic critical diameter $D_{cr}$ (nm)	Domain magnetization $M_d$ (kA m^−1^)	Anisotropy constant $K$ (kJ m^−3^)	Density $\rho$ (kg m^−3^)
Barium-ferrite	9^a^	300^f^	300–330^g^	5280^g^
		380^g^		
Cobalt-ferrite	10^b^	425^g^	180–200^g^	4907^g^
	14^c^		120^j^	5290^n^
Iron	16^d^	1707^d^	48^d^	7870^d^
	20^e^			
Iron-cobalt	16^b^	1815^d^	1.5^h^	8031^d^
	24^d^	1790^h^	15^d^	8140^h^
Iron-platinum	3^b^	1140^i^	206^f^	15,200^h^
	10^d^		7000^k^	
Maghemite	31^b^	414^g^	4.7^l^	4600^g^
	35^d^		16^j^	
Magnetite	21^d^	446^g^	23–41^g^	5180^g^
	25^b, c^		9^d^	
Nickel-ferrite	28^c^	301^c^	−0.068^c^	5380^n^
			33^m^	

^a^Estimated ($$D_{cr} \approx (150k_{B} T/\pi )^{1/3}$$ [150]), ^b^ [151], ^c^ [152], ^d^ [61], ^e^ [153], ^f^ [154], ^g^ [91], ^h^ [100], ^i^ [155], ^j^ [156], ^k^ [157], ^l^ [158], ^m^ [159], ^n^ [160]

There are several factors affecting treatment outcome of MFH [92]. Different types of material offer different heating capabilities. The size of the nanoparticle’s core and its hydrodynamic size affect the generation and distribution of heat. The magnetic property of material, the anisotropy constant affects the heat generation process. Dynamic viscosity of the medium influences the amount of heat generated by Brownian motion. The variation in the applied magnetic field strength and frequency changes the magnitude of heat generated by superparamagnetic nanoparticles. In addition, the concentration of nanoparticles is proportional to the total amount of heat generated in the treatment. The optimization of these factors is necessary so as to acquire the maximum heating for the treatment [93]. However, it is important to note that the optimum setting in generating maximum heating outcome does not ensure if the condition is suitable to be used clinically as the optimization process is often analytical based with a number of assumptions that may deviate in real life application [94].

### Type of material

The most frequently used nanoparticles in MFH is the iron oxide (maghemite or magnetite) because it is found tolerable by the human body and it is immuno-evasive [95]. An immuno-evasive behavior of particles is required for MFH to avoid unwanted immune response where a small amount of the nanoparticles may be taken up by surrounding healthy tissue. Iron oxide nanoparticles will be eaten by macrophages or neutral granulocytes and they will be degraded in lysosomes. The degraded form can be integrated into the normal iron metabolism and eliminated from the body eventually [96]. In addition, the iron oxide is able to generate enough power to induce MFH. Hence, it is an excellent biomaterial for hyperthermia [97].

Other than iron oxide nanoparticles, several alternative materials with better heating properties have been explored by researchers. By comparing the heating power of iron-cobalt and ferrite core–shell nanoparticles, it was found that iron-cobalt nanoparticles had a considerably higher specific loss power due to its high saturation magnetization [98]. The higher power dissipation reduces the required concentrations of nanoparticles to be injected in MFH. However, the toxicity of cobalt can be an issue and further processing such as coating using biomaterial is required. The comparison of more types of materials has been carried out where 6 types of nanoparticles, i.e. magnetite, maghemite, iron-cobalt, iron-platinum, barium-ferrite, and cobalt-ferrite, were compared [99]. It was shown that due to either the low rate of temperature change generated by the barium-ferrite and cobalt-ferrite or the large rate of temperature change produced by iron-cobalt; these materials were not recommended to be used in MFH. The study suggested that the magnetite, iron-platinum, and maghemite were suitable options in MFH because they would generate high enough heating power. In another study, it was found that iron-platinum nanoparticles have the capability to be the high-performance nanoheaters for their high saturation magnetization and high chemical stability [100]. At lower frequency, the iron-platinum nanoparticles provide a higher heating rate as compared to the magnetite. Since iron-platinum nanoparticles offer a superior heating power as compared to iron oxide nanoparticles, the amount of administered iron-platinum nanoparticles in generating the same quantity of heat is lower. In addition, the diameter of iron-platinum nanoparticles required to produce a higher heating rate is smaller as compared to the magnetite. This small diameter may increase the selectivity of nanoparticles in targeting the tumor attributable to its higher surface to volume ratio for functionalization on particles’ surface. Thus, the iron-platinum nanoparticle seems to be a good alternative material used for hyperthermia. More studies have to be done to test the nanotoxicity of these alternative materials before it can be deemed as a safe material to be injected into the human body.

### Particle size

The particle size influences SLP outcome significantly [97, 101–103]. Numerous studies tried to determine the optimum size of nanoparticles that would generate an optimum heating outcome. However, different studies always came out with different conclusions. For instance, for the size of magnetite, different optimum diameters had been proposed, such as 10 nm [104], 11.2 nm [105], 12 nm [106], 16 nm [103], and 19 nm [107]. Differences in the finding of optimum particle size used for the treatment are due to the differences of heating process setting; the magnetic field strength and frequency. Even the comparison was done by using intrinsic loss power (ILP), the results obtain will also be different across different studies, because of variation in the other parameters such as the anisotropy [86]. In order to get the real maximum heating, the particle size together with all other factors has to be optimized.

### Anisotropy

The magnetic anisotropy influences the hyperthermia treatment outcome [83, 108]. Through modeling, it was revealed that the nanoparticle size at which the particle generated maximum heating power decreased as the anisotropy constant increased [98]. The approximation of optimum anisotropy used to generate optimum heating can be calculated analytically using proposed formulation by Carrey et al. [86]. This anisotropy constant can be altered through modifying the methods for synthesizing nanoparticles. The shapes of nanoparticles also affected the magnetic anisotropy. Coating the nanoparticles in order to gain additional functions may also change the anisotropy constant of the particles [109].

### Viscosity

Types of medium also affect the treatment outcome. Using the same concentration of material and the same magnetic field conditions, the temperature was observed to rise highest in the water, followed by glycerol and the temperature rose lowest in collagen [110]. This phenomenon occurred because of the high viscosity of collagen that reduced the Brownian relaxation effect. Another similar experimental study was done and concluded that the heating ability of nanoparticles was reduced with an increment in viscosity as a result of the reduction in the heating contribution from the Brownian relaxation [111]. Thus, it was anticipated that due to the high viscosity of the extracellular matrix in a biological tissue, the heat loss by Brownian relaxation is negligible. This inhibition of Brownian relaxation was further proved in the experiment carried out in vitro on living cells [112]. In their study, a range of different nanoparticle types was inserted into the cell cultures. Despite different morphologies and characteristics of nanoparticles, all samples showed a decline in the heating power when the nanoparticles were attached to the cell membrane. When the particles were inside the cells, the SLP dropped by half or even to its one-tenth.

### Magnetic field strength

Magnetic field strength affects the temperature rise significantly [113]. The range of commonly used magnetic field for MFH is between 0 and 15 kA m^−1^ [64, 114]. The increment in magnetic field strength would raise the temperature of a tumor and reduce the treatment duration [115]. This relationship between magnetic field strength and generated heat was explored in a number of experiment and simulation works and through those studies, it was found SAR varies linearly with the square of magnetic field strength [107, 116].

### Magnetic field frequency

Typical magnetic field frequency used in MFH is between 0.05 and 1.2 MHz [64, 114]. It has been found that frequency and SAR have a linear relationship [107, 116]. It may be beneficial to apply a high magnetic field strength and frequency in order to produce a large amount of heat, but using high magnetic frequency and field strength on the human body may cause side effects and hence, there is a threshold that should not be exceeded. Based on an experimental study, the tolerable limit of applying the magnetic field on the human body was up to the magnetic field intensity of 35.8 A turns m^−1^ at a frequency of 13.56 MHz [117]. It was recommended that the product of magnetic field strength and frequency used in the clinical setting should not go beyond 4.85 × 10^8^ A turns m^−1^ s^−1^. However, this limit might be too stringent and a higher threshold can possibly be accepted [118]. More studies have to be done to clarify the maximum allowable magnetic field strength and frequency at different parts of the human body.

### Concentration

The total amount of heat generated in MFH and the toxicity effect on cancer cells strongly depend on the concentration of nanoparticles [119]. As the concentration of nanoparticles increases, the temperature reached by a tumor increases [113, 120]. In the experiment using the collagen to represent the tissue behavior, it was found that by doubling the concentration of particles, the temperature rise was also doubled [110]. The increment in concentration heightens the temperature by increasing the amount of heat source but it does not influence SAR generated by each nanoparticle [116].

### Polydispersity

Synthesizing monodisperse particles is extremely challenging and thus for real life application, the polydispersity of particle diameters has to be taken into consideration in calculating optimized heat generation. Studies have been conducted to observe the effect of polydispersity on the heat generation by magnetic nanoparticles. By incorporated polydispersity into heat generation equations, as shown in Table 1 Eqs. 10 and 11, it was found that the large size distribution of particles reduced the heat production of the system [91]. Experimental data have shown similar finding where the heating rate was dependent on the polydispersity and by having a lower polydispersity of nanoparticles, the heating outcome generated was substantially higher [105, 121]. A proposed method to reduce the detrimental effect of size distribution is to have an optimum anisotropy which could increase the heating power [86].

## Bioheat transfer model for heat distribution

In the nanoscale world, certain well-known Physics of phenomena behave differently. For heat transfer phenomenon, the conventional heat transfer model by conduction is no longer valid when the mean free path of material is below a threshold value and the ballistic energy transfer is more appropriate. This threshold is the mean free path of material. Rabin showed that the conventional heat transfer as defined by the Fourier law is applicable for nanoparticles with the size larger than 0.3 nm [122]. So for the heat distribution analysis in biological tissue during MFH, the Fourier law is applicable. It is important to note that there is a minimum size of the domain occupied by nanoparticles needed to achieve the hyperthermic temperature. Rabin proposed that a minimal region with a diameter of 1.1 mm occupied by nanoparticles was required to increase the temperature of tissue by 6 °C for hyperthermia [122].

In describing the heat transfer phenomenon, it is crucial to remember that the blood flow plays a significant role for dissipating heat away from tissue with the purpose of maintaining the homeostasis of the body [123]. When heat is applied to the biological tissue, there is a maximum temperature a tissue can achieve due to heat loss by the blood flow. Without the blood flow, the temperature of tissue will increase linearly with the heating time at a specific heating rate. In the presence of blood flow, the increase of temperature is nonlinear due to the heat loss and its rate of temperature rise is decreasing until it reaches the steady state. Therefore, the bioheat transfer model is established to take into account the heat loss term due to the blood flow for describing heat transfer in biological tissue [124–126].

The development of bioheat transfer equation can be traced back to more than 60 years ago when it was first proposed by Pennes [127]. Pennes modeled the heat transfer in a resting human forearm by presenting extra terms to represent the heat generation due to the metabolism in tissue and the heat transfer between the blood and adjacent tissues as shown in Table 3 Eqs. 12–14. Due to the assumption made by Pennes, the equation developed has ignored or simplified certain features of the system and consequently, the solution may have only limited real life application. Pennes equation failed to account the directional convective phenomena and the heat exchange between closely packed countercurrent blood vessels. Hence, various modified models have been developed to overcome these weaknesses. Wulff modified the basic bioheat transfer equation by introducing the effect of flow direction [128]. Chen and Holmes classified the blood vessels into the large vessels and the small vessels and accounted their effect on heat transfer separately with modified form of Pennes bioheat transfer equation as expressed in Table 3 Eqs. 15, 16 [129]. Taking into account the interaction of blood vessels, Weinbaum, Jiji and Lemons modified the model by adding the response of countercurrent heat exchange of artery and vein as presented in Table 3 Eqs. 17–19 [130–132]. A more detailed review of these modified bioheat transfer model can be found in Ref [133].

**Table 3:** Equations used in describing bioheat transfer phenomena based on basic Pennes model [133]

Eq. no.	Parameter	Equation expression	Nomenclature
	*Pennes model (1948)*		
12	Bioheat transfer equation	$\rho c\frac{\partial T}{\partial t} = - k\left[ {\frac{{\partial^{2} T}}{{\partial r^{2} }} + \frac{1}{r}\frac{\partial T}{\partial r} + \frac{1}{{r^{2} }}\frac{\partial T}{\partial \emptyset } + \frac{{\partial^{2} T}}{{\partial Z^{2} }}} \right] + q_{m} + q_{b}$	$\rho$ density of medium (kg m^−3^) $c$ medium specific heat (J kg^−1^ K^−1^)
13	Heat transfer from blood to tissue, $q_{b}$ (J m^−3^ s^−1^)	$q_{b} = (\rho c)_{b} \omega (T_{a} - T)$	$T$ tissue temperature (K) $k$ tissue specific thermal conductivity (W m^−1^ K^−1^)
14	Temperature dependent blood flow, $\omega$ (s^−1^)	$\omega = \omega_{0} (1 + \gamma T)$	$q_{m}$ heat production rate of tissue (J m^−3^ s^−1^) $q_{b}$ heat transfer rate from blood to tissue (J m^−3^ s^−1^) $r,\emptyset ,Z$ cylindrical coordinate $\omega$ blood perfusion (s^−1^) $\rho_{b}$ blood density (kg m^−3^) $c_{b}$ blood specific heat (J kg^−1^ K^−1^) $T_{a}$ arterial blood temperature (K) $\omega_{0}$ baseline of volumetric flow rate of blood (s^−1^) $\gamma$ time dependent blood flow coefficient (K^−1^)
	*Chen and Holmes model (1980)*		
15	Thermal equilibrium length, $l_{e}$ (m)	$l_{e} = \frac{{A(\rho c)_{b} \bar{V}}}{U \cdot P}$	$A$ flow area (m^2^) $\bar{V}$ local blood velocity (m s^−1^) $U$ overall heat transfer coefficient (W m^−2^ K^−1^) $P$ circumference (m) $\bar{u}$ net volume flux (m s^−1^) $k_{p}$ heat transfer coefficient of contributing vessel(W m^−2^ K^−1^) $*$ properties of large blood vessel
16	Bioheat transfer equation	$\rho c\frac{\partial T}{\partial t} = \nabla \cdot k\nabla T + (\rho c)_{b} \omega^{*} \left( {T_{a}^{*} - T} \right) - \left( {\rho c} \right)_{b} \bar{u}\nabla T + \nabla \cdot k_{p} \nabla T + q_{m}$	
	*Weinbaum, Jiji and Lemons model (1984)*		
17	Countercurrent arteries	$\left( {\rho c} \right)_{b} \pi r_{b}^{2} \bar{V} \cdot \frac{{dT_{a} }}{ds} = - q_{a}$	$r_{b}$ blood vessel radius (m) $q_{a}$ heat loss rate at artery wall (J m^−1^ s^−1^) $q_{v}$ heat gain rate at vein wall (J m^−1^ s^−1^) $g$ blood bleed off rate (m s^−1^) $T_{v}$ venous blood temperature (K) $n$ vessel number density (m^−2^) $s$ direction along a blood vessel
18	Countercurrent veins	$\left( {\rho c} \right)_{b} \pi r_{b}^{2} \bar{V} \cdot \frac{{dT_{v} }}{ds} = - q_{v}$	
19	Bioheat transfer equation	$\rho c\frac{\partial T}{\partial t} = \nabla \cdot k\nabla T + 2n\pi r_{b} \left( {\rho c} \right)_{b} g\left( {T_{a} - T_{v} } \right) - n\pi r_{b}^{2} \left( {\rho c} \right)_{b} \bar{V} \cdot \frac{{d\left( {T_{a} - T_{v} } \right)}}{ds} + q_{m}$	

## Analytical modeling

With the aim of obtaining the temperature distribution of the hyperthermia process in the biological system, the heat transfer phenomenon is modeled using the bioheat heat transfer equation. Several attempts in deriving analytical solutions of hyperthermia phenomena have been carried out. Analytical solutions were commonly obtained by simplifying problems so that exact solutions can be obtained.

Andrӓ et al. derived equations to describe the temperature distribution of MFH with the assumption that the nanoparticles samples were in the form of a sphere shape located inside the tumor [134]. Several boundary conditions were applied: firstly, the center of sphere had to be finite at all time; next, the temperature at the radius of infinity was equal to the initial temperature at all time; thirdly, the temperature distribution was assumed to be continuous at the interface between the tumor and the surrounding tissue at all time; and lastly, the heat flux was assumed to be continuous across the interfaces. Based on these boundary conditions, an analytical solution was derived to describe the temperature variation with respect to time and space. The authors further compared the derived solution with the experimental data and found an agreement between them. This analytical solution was further verified by Sawyer et al. who carried out a study on the heat distribution and found an agreement between their experimental data and Andrӓ et al’s solution [135]. However, it is important to note that the solutions of heat transfer equations derived did not consider the effect of heat loss caused by the blood flow which might affect the heat transfer process significantly in highly perfused tissues.

An improved analytical solution took into account heat loss due to the blood flow in MFH was carried out by Bagaria and Johnson [136]. In their work, the developed model consisted of 2 spherical regions where the inner region represented the tumor with nanoparticles embedded in it and an outer spherical region represented the healthy surrounding tissues. The Pennes’ bioheat transfer was applied to the model with boundary conditions including a finite temperature in the tumor, continuous temperature and heat flow between the interface of 2 spheres, the temperature of the outer sphere was equal to the body temperature and the initial temperature throughout both regions were equal to the initial body temperature. Analytical solutions were derived using separation of variables method with assumptions that the heat generation had a quadratic spatial variation and higher order polynomials deemed to be impractical to be used.

## Numerical modeling

Because of numerous assumptions used in deriving the analytical solution, the results may have oversimplified the real phenomena and have questionable accuracy. The complex properties of biological tissues, the human body regulation and the interaction between the biological environment and foreign particles are too complex to be solved analytically. Numerical methods in the modeling of the heat transfer phenomena can be useful methods to solve this complex problem. Most of the simulations of MFH heat transfer were carried out by using either the finite element method (FEM) or the finite difference method (FDM).

Sawyer et al. [135] applied FEM to model the heat transfer process in hyperthermia by using difference approximation method of the differential heat equation. The model described the heat distribution using iron-cobalt as magnetic nanoparticles in MFH for the fat and muscle tissues. The results showed the proposed model fitted with derived analytical solutions by Andrӓ et al. [134] well. Pearce et al. [137] carried out the simulation using FEM to model the heat transfer from nanoparticles to surrounding biological tissue in order to investigate the required power density to be generated by nanoparticles in hyperthermia. They showed that the tendency of particles to cluster together in vivo reduced the required power density. Pavel and Stancu applied FEM in simulating heat transfer in MFH and demonstrated that the achievable temperature in the tumor decreased considerably in the presence of blood vessel near the tumor [138]. They also showed that a smaller dose is required to reach the hyperthermic temperature in a larger tumor as compared to a smaller size tumor. The heat transfer due to the different injection sites in multiple tumor regions was simulated by Pavel and Stancu in another study [139]. By modeling the biological system using FEM software, they showed that the treatment outcomes were varied for according to the tumor types and sizes of blood vessels that presented near to the tumor. This study suggested the required concentration of nanoparticles in the hyperthermia based on the diameter of metastases in order to generate the optimum heating power. To further explore the effect of the blood vessel on heat distribution in MFH, Yue et al. [140] carried out a study using FEM to find out the influence of the vessel bifurcation on heat transfer in MFH. The study found that a larger blood bifurcation caused higher heat loss to the blood and hence, it exerted a higher cooling effect on temperature distribution. This study confirmed that the MFH treatment planning should take into account the shape, position, and size of the vessel bifurcation. Nabil et al. [141] showed the effect of blood flow and capillary on the distribution of nanoparticles and the temperature distribution generated. Miaskowski and Sawicki implemented FEM to mimic a more realistic heat transfer in the biological tissue by taking into consideration effects of metabolic heat, blood perfusion, and convection skin cooling [126]. Their study simulated the hyperthermia in breast cancer and showed that the proposed model could potentially be used to provide an accurate temperature mapping of MFH.

Another well-known numerical method commonly used in the biomedical simulation is FDM. Chen et al. believed that FDM was an effective method to be used in the modeling of MFH heat transfer phenomena for solving the three-dimensional non-homogenous problem in hyperthermia [142]. Rast and Harrison simulated the heat transfer of MFH using FDM [114]. In the model, they coupled the heat production using the Maxwell’s equation with the Pennes’ bioheat transfer equation. The domain created was a multilayer domain with the innermost layer consisted of the tumor and nanoparticles, covered by the gray matter which was further enclosed by the perfused dermal and skeletal layers. The nanoparticles were assumed to be distributed homogeneously all over the tumor. The results showed that blood perfusion rate affected the temperature rise in the tumor. Another FDM simulation was carried out by Bagaria and Johnson [136]. In this work, the numerical model was developed based on finite differencing scheme and it showed a good agreement with the derived analytical solution. Craciun and Calugaru also explored the use of FDM in describing the heat transfer in MFH [143]. They created the algorithm for solving the heat transfer equation by incorporating the tissue metabolism heat term and the heat sink due to the blood vessel. To improve the performance of the model, new technology or system can be introduced into the numerical modeling, such as the use of the parallel computing to enhance the processing speed [144].

## Optimization model

Optimization of hyperthermia process is crucial in the clinical setting for the purpose of maximizing the therapeutic effects and minimizing any side effects. Besides, optimization of MFH should ensure that the required amount of nanoparticles is as small as possible while the nanoparticles are still able to produce high enough heating power. Hence, by carrying out optimization, the limit of targeted area can be controlled, the potential overheating may be avoided and any harm to the surrounding healthy tissue may be minimized.

The optimization of MFH heating process can be found in the study done by Bagaria and Johnson [136]. In this study, first, the domain was modeled as 2 concentric spheres to represent the tumor and adjacent tissue and the Pennes’ bioheat transfer equation was applied. The optimization was done with objectives to sustain the therapeutic temperature in tumors and keep adjacent healthy tissue at an acceptable temperature range as shown in Table 4 Eq. 20. A penalty function was applied in the optimization which minimized both the insufficient heating of tumor and the overheating in the normal tissue. Using this model setting, the authors concluded that for a fixed amount of nanoparticles, optimization of particle distribution in the tumor can improve the therapeutic effect on tumor and maintain the safety heating limit in the surrounding healthy tissue. A similar model setting was imposed in the study done by Mital and Tafreshi [145]. However, there was a slight difference in the optimization function used. Instead of just focusing on minimizing the error, the optimization function rewarded the therapeutic effect on a tumor. In the other words, it was favorable for the tumor to be heated to the range of therapeutic temperature while minimizing the harmful effect on surrounding tissue. The optimization was set by maximizing a fitness function expressed in Table 4 Eq. 21 which value increased if there was a higher damage in the tumor and decreased if the treatment caused more harm to adjacent healthy tissue. Based on the available power dissipation records, the results of this optimization suggested the optimum treatment time of hyperthermia was 1 h. Another optimization of MFH heating process was done by Salloum et al. [146]. Salloum et al. simulated the heat transfer pattern using FEM in order to obtain optimum parameters for hyperthermia with multiple nanoparticle injection sites. The tumor was designed as an irregular tissue in a large cubical domain. The objective function was set to ensure that at least 90% of the tumor had a temperature rise to 43 °C while less than 10% of the surrounding healthy tissue hit the temperature of 43 °C or higher as shown in Table 4 Eq. 22.

**Table 4:** Summary of optimization model for MFH

Eq. no.	Parameter	Equation expression	Nomenclature
	*Bagaria and Johnson optimization model*, 2005 [136]		
20	Objective function, $f$ Error function for insufficient and overheating in tumor, $E_{1}$ Error function for overheating in healthy tissue, $E_{2}$	$f = \sqrt {E_{1} + E_{2} }$ For $42 \le T_{1\infty } \le 45$ $E_{1} = 0$ For $T_{1\infty } < 42$ or $T_{1\infty } > 45$ $E_{1} = W_{1} \mathop \sum \nolimits (T_{1\infty } - 42)^{2}$ For $T_{2\infty } = 37$ $E_{2} = 0$ For $T_{2\infty } \ne 37$ $E_{2} = W_{2} \mathop \sum \nolimits (T_{2\infty } - 37)^{2}$	$T_{1\infty }$ steady state temperature of a point in tumor $T_{2\infty }$ steady state temperature of a point in healthy tissue $W_{1}$ weight factor for insufficient heating in tumor $W_{2}$ weight factor for overheating in healthy tissue
	*Mital and Tafreshi optimization model*, 2012 [145]		
21	Objective function, $f$ Thermal damage, $D$	$f = \propto_{1} D_{1} - \propto_{2} D_{2}$ $D\left( {x,y} \right) = \mathop \smallint \limits_{0}^{{t_{f} }} R^{{(43 - T\left( {x,y,t} \right))}} {\text{d}}t$	$\propto_{1}$ weight factor for tumor damage $\propto_{2}$ weight factor for healthy tissue damage ${\text{D}}_{1}$ thermal damage value of tumor ${\text{D}}_{2}$ thermal damage value of healthy tissue $t_{f}$ period of time $R$ empirical constant $T$ temperature at a point at a time $t$ time
	*Salloum, Ma and Zhu optimization model*, 2009 [146]		
22	Objective function, $f$ Error function for deviation of tumor boundary temperature, $E$ Percentage of tumor volume with $T \ge T_{n}$, $R_{1}$ Percentage of tumor boundary with $T \ge T_{n}$, $R_{2}$	$f = \frac{E}{{R_{1} \cdot R_{2} }}$ $E = \frac{{\mathop \sum \nolimits \left( {T_{b} - T_{n} } \right)^{2} }}{{\left( {T_{a} - T_{n} } \right)^{2} }}$ $R_{1} = \frac{{V_{{{\text{tumor}}, T \ge T_{n} }} }}{{V_{{{\text{tumor}},{\text{total}}}} }} \cdot 100$ $R_{2} = \frac{{S_{{{\text{boundary}}, T \ge T_{n} }} }}{{S_{{{\text{boundary}},{\text{total}}}} }} \cdot 100$	$T_{b}$ temperature at tumor boundary $T_{n}$ cutoff temperature where the value above it is desired for tumor and below it for healthy tissue $T_{a}$ arterial blood temperature $V$ volume $S$ surface area

In order to receive clinical acceptance, the results obtained from simulation (analytical, numerical and optimization model) must be verified with real clinical data. However, a major hindrance in the verification of these methods is an unavailability of accurate clinical data. The temperature distribution measurement comes with errors especially for temperature measurement of a deep-seated tumor. The significance difference in thermal conductivity of metal thermocouple and surrounding biological tissue causes distortion in temperature measurement [147]. This thermal conductivity differences issue can be overcome by applying insulation on the probe. However, the use of insulation will also cause an error in the reading due to temperature drop across the sheath wall [148]. Besides, the use of a magnetic field for heating nanoparticles may also heat up the metal thermocouples through eddy currents heating and show an unusual reading. With the aim of minimizing the error in temperature measurement, careful thermocouple probe, and instrumentation design by taking into account the use of magnetic field as nanoparticle heating method is required. Exploring new alternative temperature mapping technique may also offer a solution for obtaining higher accuracy clinical temperature reading, such as the use of magnetic resonance imaging for temperature mapping during hyperthermia treatment [149].

## Conclusion

Hyperthermia has the potential to be a new alternative therapy modality for cancer treatment. Hyperthermia selectively targets the tumor without harming surrounding tissue as the tumor has higher sensitivity to heat. A promising heating method for the hyperthermia therapy is MFH. Several simulations of heat transfer phenomena in MFH have been carried out and proposed based on the bioheat transfer equation. However, there are still needs for the further study of simulation that can mimic the biological response to the applied heat. By having a more accurate simulation, the effect of various parameters on the treatment outcome can be determined. In addition, it will allow a better control of therapy and can be used as an alternative temperature mapping technique. Further optimization studies also have to be carried out to find the optimum material used which could generate the highest heating and the optimum treatment setting that can enhance the treatment outcome.

## Abbreviations

FDM: finite difference method
FEM: finite element method
ILP: intrinsic loss power
MFH: magnetic fluid hyperthermia
SAR: specific absorption rate
SLP: specific loss power
